# Biological Effects of Chitosan-Based Dressing on Hemostasis Mechanism

**DOI:** 10.3390/polym11111906

**Published:** 2019-11-19

**Authors:** Yi-Wen Wang, Chuan-Chieh Liu, Juin-Hong Cherng, Chien-Seng Lin, Shu-Jen Chang, Zhi-Jie Hong, Cheng-Che Liu, Yaw-Kwan Chiu, Sheng-Der Hsu, Hung Chang

**Affiliations:** 1 Department and Graduate Institute of Biology and Anatomy, National Defense Medical Center, Taipei 100, Taiwan; christmas1035@mail.ndmctsgh.edu.tw (Y.-W.W.); i72bbb@gmail.com (J.-H.C.); 2 School of Medicine, Fu-Jen Catholic University, New Taipei City 100, Taiwan; chuanchiehliu@gmail.com; 3 Department of Cardiology, Cardinal Tien Hospital, Taipei 100, Taiwan; 4 Department of Gerontological Health Care, National Taipei University of Nursing and Health Sciences, Taipei 100, Taiwan; 5 Department of Emergency and Critical Care Medicine, Cheng Hsin Rehabilitation Medical Center, Taipei 100, Taiwan; jason.cslin2019@gmail.com; 6 Division of Rheumatology/Immunology/Allergy, Department of Internal Medicine, Tri-Service General Hospital, National Defense Medical Center, Taipei 100, Taiwan; belle661011@gmail.com; 7 Division of Traumatology, Department of Surgery, Tri-Service General Hospital, National Defense Medical Center, Taipei 100, Taiwan; lgf670822@mail.ndmctsgh.edu.tw; 8 Department of Physiology and Biophysics, Graduate Institute of Physiology, National Defense Medical Center, Taipei 100, Taiwan; chencheliu2002@gmail.com; 9 Department of Pediatrics, School of Medicine, National Defense Medical Center, Tri-Service General Hospital, National Defense Medical Center, Songshan Branch, Taipei 100, Taiwan; yawkwan@gmail.com; 10 Graduate Institute of Medical Sciences, National Defense Medical Center, Taipei 100, Taiwan; 11 Division of Thoracic Surgery, Department of Surgery, Tri-Service General Hospital, National Defense Medical Center, Taipei 100, Taiwan

**Keywords:** antithrombin, chitosan dressing, coagulation factor, hemorrhage, hemostasis

## Abstract

There have been numerous recent advances in wound care management. Nevertheless, the assessment of hemostatic dressing is essential to enable surgeons and other physicians and healthcare professionals to make the correct decisions regarding the disposition of severe hemorrhage. Here, we investigated the relative efficacies of chitosan-based and conventional gauze dressings in a rat model of femoral artery hemorrhage and in patients with surgical wounds. Dressing effectiveness was evaluated based on hemostatic profiles, biocompatibility, antimicrobial activity, and blood factor responses in coagulation. Relative to standard gauze dressing, the chitosan fiber (CF) dressing treatment significantly shortened the time to hemostasis in injured rats. Moreover, the CF dressing significantly prolonged partial thromboplastin time, enhanced blood absorption, and reduced antithrombin production without altering the prothrombin ratio. Unlike regular gauze bandages, the CF dressing demonstrated remarkable antibacterial activity. The results of this study indicate the effectiveness of chitosan as a hemostatic dressing and elucidate its underlying mechanism. It is possible that chitosan surgical dressings could serve as first-line intervention in hospital emergency care for uncontrolled hemorrhage.

## 1. Introduction

Serious hemorrhage caused by abnormal blood coagulation, injuries, ruptured organs, surgical complications, femoral artery puncture site bleeding, or vascular malformation may induce potentially fatal shock and extreme local suppression [1,2,3]. To prevent death caused by bleeding, effective hemorrhage control must be provided immediately after the injury and should control active bleeding within minutes. Several advanced hemostatic dressings have been recently applied to help staunch massive bleeding [4,5,6]. Chitosan-based dressings effectively suppress the hemorrhage. They are easy and simple to use, may be precisely applied to deeply penetrating wounds, and could potentially stimulate regeneration [7]. However, the underlying mechanism by which chitosan induces rapid clotting is unclear. It merits further investigation as it furnishes effective hemorrhage management strategies that could save lives at the acute emergency level.

Chitosan is a commercially available polysaccharide prepared by controlled chemical chitin de-*N*-acetylation. It is composed of randomly distributed *D*-glucosamine and *N*-acetyl-*D*-glucosamine residues that confer a pH-dependent positive charge to the polymer. A previous study showed that the positive charge on chitosan promoted erythrocyte adhesion, fibrinogen adsorption, and platelet adhesion and activation but also inhibited contact system activation [8]. Along with its wound-healing promoting effect, chitosan has hemostatic and antihyperlipidemic activity. It stimulates immunity, promotes mucoadhesion, is permeable to oxygen, and has antibacterial efficacy [9,10,11]. The hemostatic property of chitosan was ascribed to its polycationic content and nonspecific plasma membrane binding. It has positively charged amino groups along its molecular chains [12].

Hemostasis occurs in the first stage of wound injury. It is manifested by wound closure via coagulation. The key role of coagulation is to control bleeding by converting soluble fibrinogen proteins into insoluble fibrin and forming a lattice over the wound via intrinsic and extrinsic pathways [13]. Several studies demonstrated that chitosan activates or enhances various coagulation factors that accelerate hemostasis [14,15,16]. However, the underlying mechanism involved is poorly understood. The response of blood elements to chitosan during coagulation and the safety and efficacy of this biomaterial at the onset of bleeding must be assessed. Here, we compared the relative efficacy of two different chitosan-based dressings and regular gauze at staunching massive bleeding in a rat femoral artery hemorrhage model and controlling bleeding from surgical wounds. The aim of the present study was to evaluate the effectiveness of chitosan-based dressings at (1) accelerating clotting, (2) preventing bacterial infection, and (3) promoting blood coagulation factor responses.

## 2. Materials and Methods

The present study comprised four steps: (1) Characterization of chitosan-based dressing; (2) evaluation of the efficacy of chitosan dressing at accelerating clotting time and staunching massive bleeding in a rat femoral artery hemorrhage model; (3) *in vitro* analysis (cell viability and antimicrobial activity) and ex vivo coagulation tests (prothrombin time (PT), activated partial thromboplastin time (aPTT), and coagulation factors) of the chitosan dressing; and (4) assessment of hemoglobin absorption by chitosan dressing applied to surgical wounds. The Animal Care Committee at the National Defense Medical Center of Taiwan, R.O.C. approved the animal study (IACUC–18–312). The clinical trial was approved by the Institutional Review Board (IRB No. 2–105–05–106) and the General Clinical Research Center (GCRC No. 106–137) of the Tri-Service General Hospital of Taipei, Taiwan. It was also entered into the US National Institute of Health Clinical Trials Registry (NCT03907111).

### 2.1. Preparation of Experimental Dressing

Two new chitosan-based dressings were used as the experimental treatments. The chitosan raw material was purchased from Une Shin Trading Co., Ltd. (CAS No. 9012–76–4; Formula: (C_8_H_13_NO_5_)_n_; New Taipei City, Taiwan, R.O.C.) with medium molecular weight of approximately 100,000 Da and 85% degree of deacetylation.

#### 2.1.1. Chitosan Fiber (CF) Preparation

CF was produced by employing a wet spinning method. Chitosan raw material was dissolved in 3% (*v/v*) and 5% (*w/v*) concentration of acetic acid by stirring overnight at 25 °C. The solution was then diluted with methanol to reach 3% (*w/v*) final solution concentration. Further, the solution was filtered through a cloth filter and placed in an ultrasonic bath to remove the air bubbles. The solution was injected into a coagulation bath maintained at 40 °C containing a 10% solution of 1 M NaOH in distilled water. The fibers were allowed to form in this medium for one day and then washed several times with distilled water. Next, they were suspended in aq. 2% TWEEN20 for 5 min and then soaked by 50%, 60%, and 70% methanol for 5 min, respectively. The chitosan filaments were compressed to drain the absorbed liquid on the mangle machine and then were dried in the oven at 60 °C in a mold. Finally, the CF was then packed in a pouch and sterilized using gamma radiation of 25 kGy.

#### 2.1.2. Chitosan Sponge (CP) Preparation

CP was fabricated by freezing and lyophilizing the chitosan solution in the Lyophilizer (FD24-4S; Kingmech Scientific CO. LTD., Taipei, R.O.C.). Chitosan solutions with concentrations of 2% (*w/v*) were prepared by dissolving the chitosan in 3% (*w/v*) of acetic acid. This solution was stirred gently for approximately 2 to 5 h at 50 °C until it became transparent and was neutralized to pH 7.4 using 1 N NaOH. The resulting square sample was 4 mm in diameter and 100 mm^2^ in area and was obtained using 15 mL of the abovementioned solution per grid, followed by cooling at 4 °C for 2 h. The subsequent cooling protocol of lyophilizer was −35 °C for 18 h and 0 °C for 24 h, followed by gradient heating at 5 °C for 1 h, 10 °C for 1 h, 15 °C for 10 min, and 20 °C for 10 min. Finally, the chitosan solution was completely lyophilized by freeze-drying at 25 °C for 24 h. The CP was then packed in a pouch and sterilized using gamma radiation of 25 kGy.

### 2.2. Physical Characteristics

#### 2.2.1. Raman Measurement

The structural characteristics of the samples were identified by Raman spectrometry (Nicolet; Thermo Fisher Scientific, Waltham, MA, USA) with an excitation wavelength of 633 nm. For each measurement, 500 scans were coded at 4 cm^−1^ resolution. The wavenumber ranged from 500–3500 cm^−1^.

#### 2.2.2. Scanning Electron Microscopy (SEM)

The surface morphology of the dressing was examined with a Hitachi S–3000N SEM (Hitachi High Technologies, Krefeld, Germany). The samples were fastened to carbon stubs and mounted on aluminum stubs. SEM images were acquired at an accelerating voltage of 1.5 kV, a working distance of ~15.0 mm, and 100×–500× magnification. Surface area of the dressings was measured according to SEM micrographs using ImageJ software (*n* = 5).

### 2.3. Water Absorptivity Determination

Water absorptivity characteristics of the dressings were determined by gravimetric approaches [17]. For water absorption test, each dressing was cut into 4 cm × 4 cm test pieces. All samples were oven-dried at 40 °C for 24 h and immediately weighed (*W*_ovendried_). Next, each dressing was immersed in distilled water at 25 °C for 24 h, wiped with a dry cloth, and immediately weighed (*W*_wet_). For moisture content test, the test pieces were directly weighed (*W*_stock_) and compared to its weight after being dried (*W*_ovendried_) for calculating the content. Water absorption and moisture content of dressings were calculated as follows:

Water absorption ratio (%) = (*W*_wet_ − *W*_ovendried_)/*W*_ovendried_ × 100 (1)

Moisture content ratio (%) = (*W*_stock_ − *W*_ovendried_)/*W*_stock_ × 100. (2)

### 2.4. Animal Model of Femoral Artery Hemorrhage

SD rats weighing ~350–400 g (*n* = 21) were fasted for 12 h before surgery. They were randomized for gauze, CF, and CP dressings. Before surgery, they received intramuscular injections of tiletamine and zolazepam (25 mg kg^−1^ + 25 mg kg^−1^, respectively) and xylazine (5 mg kg^−1^). After they were shaved and their skin was sterilized, their femoral arteries were identified, partially exposed by ~1 cm, proximally and distally occluded with vascular clamps, and perforated at the anterior surface with surgical scissors. The vascular clamps were then removed to allow 10 s free bleeding.

### 2.5. Wound Treatment and Hemostasis Time Analysis

All arterial injuries, dressing applications, and compressions were performed by the same investigator (S.D. Hsu) to minimize variability. After 5 min compression per group, hemostasis was observed for 3 min without removing the dressing. If initial bleeding was not controlled, the dressing was removed and replaced with a new one. Compression was repeated for 2 min and hemostasis was observed for the next 3 h. Stability of the hemostasis provided by the dressings was tested by flexing and stretching the wounded legs of the surviving animals 5× to simulate walking. At the end of the experiments, the dressings were gently removed from the wounds to examine the hemostatic clots and artery patency.

### 2.6. Cell Viability

Each dressing was placed in direct contact with a cell culture to assess biocompatibility. Human skin fibroblast cells (WS1, ATCC number: CRL–1502) were cultured in Dulbecco’s modified Eagle’s medium (DMEM) supplemented with 10% (*v/v*) fetal bovine serum and antibiotics (1 U mL^−1^ streptomycin–penicillin). Cells were plated at 10^5^ cells mL^−1^ in 24-well plates and incubated in a humidified incubator at 37 °C. under 5% CO_2_. Cell viability was analyzed by the 3-(4,5-dimethylthiazol-2-yl)-2,5-diphenyltetrazolium bromide colorimetric (MTT) assay. Cells incubated without the dressing material samples served as the control. After 24 h and 48 h incubation, the medium from each group was removed and replaced with 20 μL MTT (5 mg mL^−l^) and incubated at 37 °C for 4 h. The supernatant was carefully removed and dimethyl sulfoxide (DMSO) was added to each well to dissolve the crystals by gentle agitation for 10 min. The absorbance of each well at 570 nm was read on a microplate reader (Bio-Tek ELX-800; BioTek, Winooski, VT, USA). The tests were performed in triplicate.

### 2.7. Antimicrobial Test

The dressings were cut into suitable sizes for testing and sterilized under UV light at 25 °C for 12 h. Separate agar plates were inoculated with 1–5 × 10^8^ CFU mL^−1^ *Staphylococcus aureus*. The samples were placed onto the inoculated agar surface with sterilized tweezers. The samples were uniformly embedded into the agar. The petri dishes were incubated at 37 °C for 24 h and 72 h and immediately examined for bacterial growth. The Adenosine triphosphate (ATP) bioluminescence assay expressed relative luminescence unit (RLU) to assessment per sample by ATP luminometer (LuciPac Pen PD 30, Kikkoman Biochemifa Co., Tokyo, Japan).

### 2.8. Measurement of Hemoglobin Absorption by Dressing Applied to Patients with Surgical Wounds

Patients undergoing abdominal surgery had their incisions swabbed with the dressing materials for 3 min, 5 min, and another 5 min of manual compression. The dressings were collected and stored in transparent jars containing 0.9% (*w/v*) normal saline for 1 min, 3 min, 5 min, and 10 min. One milliliter of the solution was extracted from the dressing and its optical density was measured at 540 nm wavelength. The hemoglobin concentration was determined by interpolation from a hemoglobin standard (H7379, Sigma-Aldrich; Merck KGaA, Darmstadt, Germany). The experiment was performed in triplicate.

### 2.9. Prothrombin Time (PT), Activated Partial Thromboplastin Time (aPTT), and Coagulation Factor Assays

Peripheral whole blood was collected from healthy volunteers and transferred to vials containing 3.2% (*w/v*) sodium citrate. For the PT and aPTT tests, the blood was incubated with each biomaterial for 10 min, 30 min, and 60 min, respectively. After incubation, the dressings were removed and the sera were collected and subjected to an automated blood hemostasis analyzer (CS-2100i; Sysmex Corp., Kobe, Japan). Each experiment was repeated in triplicate. For the coagulation factor analysis, the blood was incubated with each dressing for 30 s, 1 min, 3 min, and 10 min, respectively. The sera were analyzed with a multiplex immunoassay kit measuring coagulation protein targets using Luminex xMAP technology (Coagulation 6-Plex Human ProcartaPlex Panel 1 (EPX060–10824–901; factor XI, factor XII, factor XIII, antithrombin, prothrombin, CRP) and Coagulation 3-Plex Human ProcartaPlex Panel 2 (EPX030–10823–901; factor V, factor VII, factor VIII) (Thermo-Fisher Scientific, Waltham, MA, USA)). The Luminex multiplex immunoassay procedure was performed according to the manufacturer’s instructions. Sera were assayed in duplicate and incubated for 18 h. The sera were added to 96-well plates containing antibody-coupled beads. The plates were run on a Bio-Plex 200 system (Bio-Rad Laboratories, Hercules, CA, USA) and examined with Bio-Plex Manager v. 61 (Bio-Rad Laboratories, Hercules, CA, USA).

### 2.10. Data Analysis

Data are means ± standard error of the mean (SEM). Treatment means were compared by one-way ANOVA. *p < 0.05, **p < 0.01, ***p < 0.001 were considered statistically significant.

## 3. Results and Discussion

### 3.1. Characterization of Chitosan-Based Dressings

**Figure 1:**
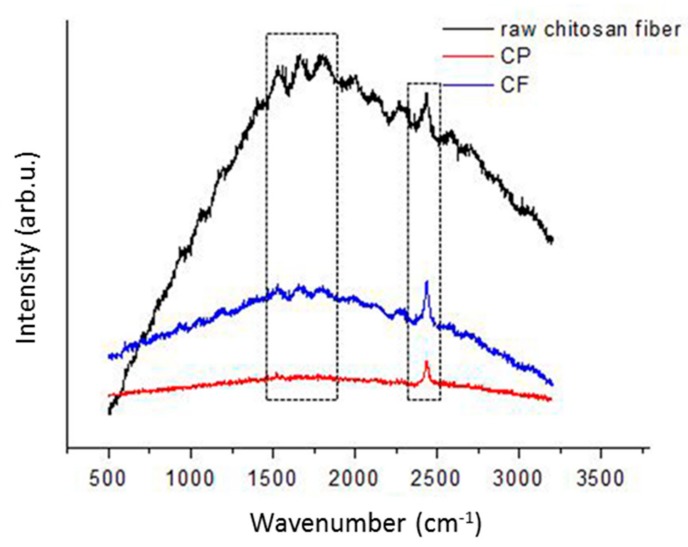
Raman scattering spectrum of chitosan in raw chitosan fiber, chitosan fiber (CF) dressing, and chitosan sponge (CP) dressing. The left rectangle included C–N–H and C=O bond group, the right rectangle indicated O–H carboxylic bending.

Raman spectroscopy was performed to identify the structural characteristics of chitosan in CF and CP dressings compared to those of unmodified raw chitosan fiber. As shown in Figure 1, raw chitosan fiber generated vibrational lines at 1540 cm^−1^, 1667 cm^−1^, 1805 cm^−1^, and 2434 cm^−1^, which corresponded to C–N–H, C=O, and O–H carboxylic bending vibrations, respectively, and these were the main indications of the presence of chitosan [18,19,20]. Both CF and CP dressings display these vibrational characteristics but at lower intensity. Variations in vibrational line intensity are associated with alterations in the molecular properties by close packing in different states of the material.

**Figure 2:**
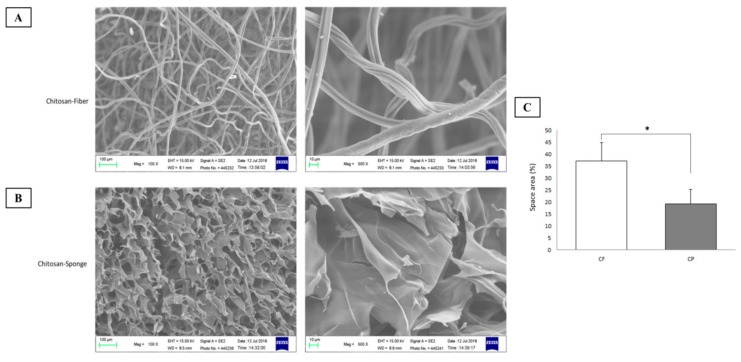
Morphology of chitosan-based dressings. (**A**) Chitosan fiber dressing. (**B**) Chitosan sponge dressing. (**C**) Surface area of dressings. (CF, chitosan fiber; CP, chitosan sponge), *p < 0.05.

Scanning electron microscopy (SEM) was performed to disclose the morphology of each type of chitosan dressing (Figure 2). The surface of the CF dressing was uniformly fibrous. It consisted of fibers ~10 μm in diameter. The surface of the CP dressing was highly porous. Its pores had a diameter range of ~50–100 μm. Both dressings had large surface areas and were composed of interconnected networks, and the CF dressing displayed the highest surface area. This structure is excellent for wound treatment as it absorbs drainage, allows oxygen permeation, and prevents bacterial infection [21]. In this way, it can hasten hemostasis.

**Figure 3:**
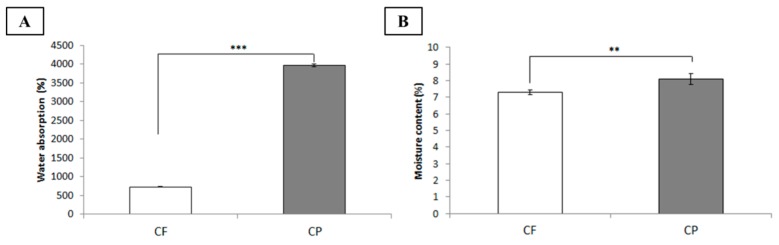
Water absorptivity of chitosan-based dressings, ***p < 0.001. (**A**) Water absorption. (**B**) Moisture content. (CF, chitosan fiber; CP, chitosan sponge), **p < 0.01.

The ideal dressing provides a moist environment that supports the penetration of therapeutic agents and has high absorptive capacity. It must maintain a wet environment, which is essential for the protection and healing of open wounds. Here, water absorptivity characteristics (including water absorption and moisture content) were determined and compared for each dressing. After sufficient immersion, both chitosan-based dressings demonstrated remarkable water absorption (Figure 3A) possibly because of the –OH, –COC, and –NH functional groups in chitosan that form hydrogen interactions. In this study, the CP dressing had the greatest water absorption perhaps because lyophilization process increased its pore size relative to the other materials [22]. This modification enlarged its voids, enhanced its capillarity, and increased the amount of water it could absorb and trap. Thus, there is no doubt that the CP dressing also absorbed the highest moisture content (Figure 3B). Thus, highly porous chitosan-based dressing might protect wound from exposure to excess fluids.

### 3.2. Hemostasis

**Figure 4:**
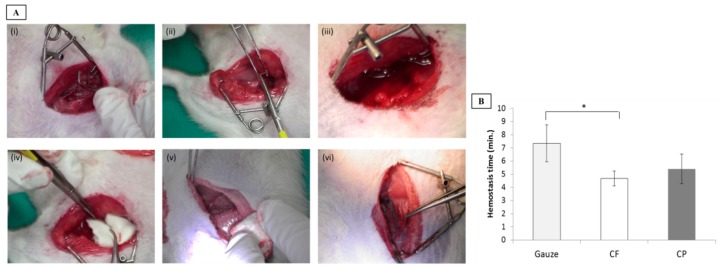
Hemostatic efficacy of dressings tested on a rat femoral artery hemorrhage model. (**A**) Schema of rat femoral artery wound treatment. (**B**) Hemostasis time analysis (CF, chitosan fiber; CP, chitosan sponge), *p < 0.05.

A rat femoral artery hemorrhage model was used to evaluate the dressings in this study. This model represents severe injury to the groin area with partial destruction of the femoral artery and life-threatening hemorrhage that regular dressings cannot control. Moreover, this trauma is not amenable to management with a tourniquet. Dressing efficacy was assessed by measuring the time required to achieve hemostasis after dressing application. Arrest of bleeding for ≥3 min after compression was considered initial hemostasis. As shown in Figure 4B, rats treated with the CF and CP dressings were hemostatic after 4.69 min and 5.40 min compression, respectively. The gauze treatment effected hemostasis within 7.33 min. The CF dressing realized hemostasis significantly faster (*p* = 0.045) than the gauze. Compared to CP dressing, CF dressing furnished a larger surface area and more effectively simulated the ECM fiber structure. Therefore, it could make comparatively closer contact with the blood in the wound.

Chitosan-based dressings, especially CF, had superior hemostatic efficacy to regular surgical dressings. Chitosan has cationic moieties such as NH_3_^+^ that have a positive effect on hemostasis. Once the chitosan dressing attaches to the wound, it forms metal cation–chitosan complexes that enhance erythrocyte and platelet adhesion, create an anionic environment at the injury site, and induce agglomeration [23]. Chitosan–erythrocyte interactions form local networks independently of other hemostatic agents [24]. When the bleeding has stopped, the chitosan dressing creates an antibacterial layer to prevent wound infection and create an environment conducive to wound healing. We tested the antimicrobial properties of the various dressing materials both in vitro and on patients with surgical wounds. Going forward, we compared only the performance of CF dressing with regular gauze as the former presented with excellent hemostasis of femoral artery hemorrhage.

### 3.3. Biocompatibility Assessment

**Figure 5:**
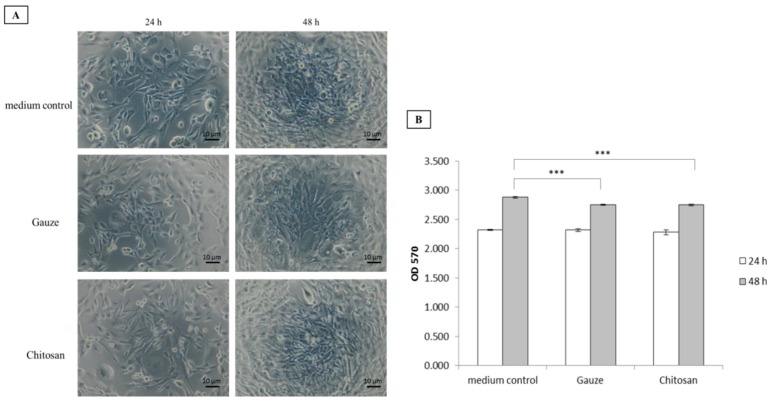
Biocompatibility of gauze and chitosan fiber dressings. (**A**) Cell morphology. (**B**) Cell viability, ***p < 0.001. (WS1 fibroblasts; 24 h and 48 h incubation).

Dressing cytotoxicity was evaluated on WS1 cells cultured with the dressing materials for 24 h and 48 h. Both types of dressings were relatively nontoxic to the WS1 cells. Further, they served as platforms for fibroblast proliferation (Figure 5A,B). Compared to the control, both dressings were equally efficacious at furnishing environments supporting cell growth and were, therefore, highly biocompatible. This assessment of biomaterials is vital as wounds are at risk of exposure to cytotoxic environments that could impede healing.

### 3.4. Antimicrobial Activity

**Figure 6:**
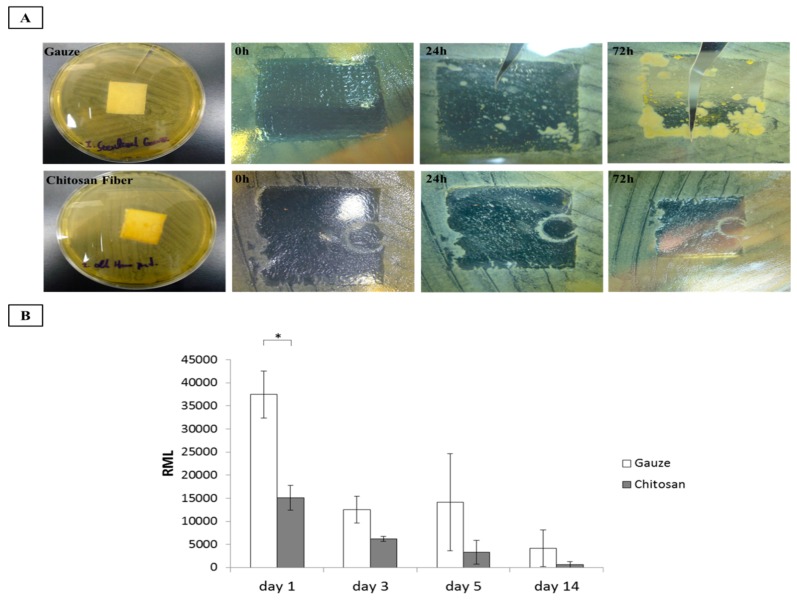
Antimicrobial activity evaluation. (**A**) *In vitro*. (**B**) Adenosine triphosphate (ATP) assay of microbial proliferation in patients with surgical wounds, *p<0.05.

Infection is a potentially lethal risk factor in uncontrolled hemorrhage. It may result either from the wound management technique or the inability of the dressing to protect the lesion from bacterial invasion. Infections may trigger systemic immune responses and inhibit several key processes involved in wound healing. In this study, we evaluated the relative antimicrobial efficacies of the wound dressings. Gauze and CF dressings were exposed to *Staphylococcus aureus* for ≤72 h and their antimicrobial activity was compared. The CF dressing was strongly antibacterial during the first 24 h and completely inhibited bacterial growth after 72 h. In contrast, the gauze demonstrated poor efficacy against *S. aureus* (Figure 6A). We performed similar antibacterial efficacy assessments on patients with surgical wounds and dressings for ≤ 14 d. As shown Figure 6B, the bacterial counts in the CF dressings on the surgical wounds were significantly lower than those in the gauze after 1 d. This trend continued over time but no statistical difference was detected between treatments. Thus, CF dressing is substantially more antimicrobial than gauze. Previous studies revealed that chitosan exhibited strong antibacterial activity [25,26]. Chitosan is a polymer with numerous basic amino groups that confer it with an overall cationic charge particularly at acidic pH. Interactions between positively charged chitosan molecules and negatively charged microbial cell membranes may cause the latter to disrupt. Therefore, the bacterial cells lyse and release their intracellular contents [27,28,29]. In this way, chitosan inhibits bacterial growth.

### 3.5. Evaluation of Hemoglobin Absorption

**Figure 7:**
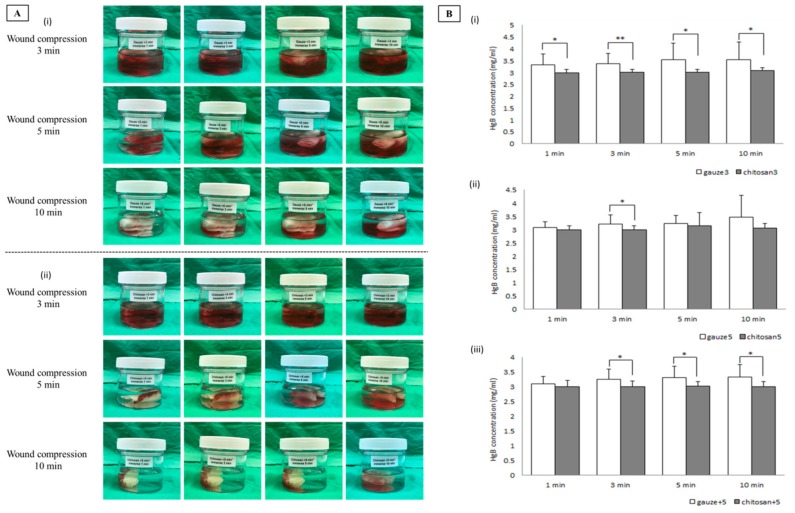
Hemoglobin absorption analysis. (**A**) Gauze and chitosan fiber dressings in contact with surgical wounds and normal saline solution. (**B**) Hemoglobin concentration in normal saline solution (1 min, 3 min, 5 min, and 10 min incubation), *p<0.05, **p < 0.01.

CF and gauze dressings were used to swab surgical incisions for 3 min, 5 min, and another 5 min of suppression. The dressings were then collected and analyzed. Figure 7Ai shows that the gauze dressing did not adequately absorb blood. At each time point, it left blood mixed with normal saline on the wounds. On the other hand, the CF dressing more effectively absorbed blood (Figure 7Aii) and left comparatively less blood and saline solution on the wound. Thus, it was better than gauze at blood absorption. To determine the relative amounts of blood not absorbed by the dressings, a hemoglobin assay was performed. At every time point, the residual hemoglobin concentration in the normal saline touching the CF dressing was significantly lower than that in contact with the gauze dressing (Figure 7Bi–iii). Electrostatic interactions between chitosan and erythrocytes form agglutination nets. Chitosan may also bind to hemoglobin and form complexes via hydrogen interactions. Chitosan alters hemoglobin microstructure and increases its viscosity [30]. These interactions account for the hemostatic properties of the biomaterial and accelerate coagulation. This investigation indicated that chitosan has remarkable pro-coagulant activity.

### 3.6. Chitosan Dressing in the Coagulation Cascade

Effective blood coagulation is essential for hemostasis. During hemostasis, an imbalance between anti-coagulation and pro-coagulation activities may have fatal consequences such as hemorrhage and thrombosis [31]. Thus, the effects of biomaterials on coagulation must be considered. In this study, PT, aPTT, and coagulation factor assays were conducted to establish the effects of chitosan on coagulation. PT and aPTT represent extrinsic and intrinsic blood coagulation pathways, respectively, and are measured to evaluate the ability of the blood to form clots. PT is commonly calculated as an international normalized ratio (INR).

**Figure 8:**
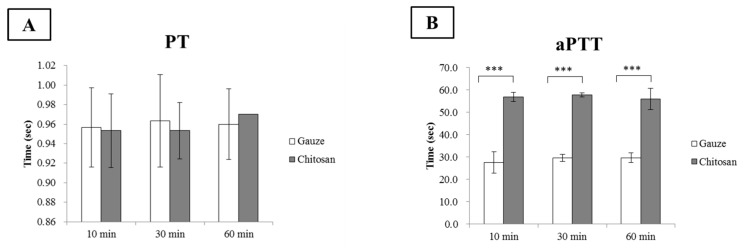
Effects of gauze and chitosan fiber dressings on (**A**) prothrombin time (PT) and (**B**) activated partial thromboplastin time (aPTT), ***p < 0.001..

The gauze dressing had INR of ~0.95, 0.96, and 0.96 for ≤10 min. However, the CF dressing had INR of ~0.95, 0.95, and 0.97 for 10 min, 30 min, and 60 min per interaction, respectively (Figure 8A). Both dressings presented with the normal INR range (0.8–1.1; [32]). Therefore, neither dressing significantly influenced the extrinsic coagulation pathways. On the other hand, the CF dressing significantly prolonged aPTT compared to the gauze dressing. The CF dressing had aPTT of ~56.9, 57.7, and 56.0 s whereas the gauze dressing had aPTT of ~27.5, 29.5, and 29.7 s at 10 min, 30 min, and 60 min per interaction, respectively (Figure 8B). In aPTT, contact system activation is the initial step in the intrinsic coagulation pathway and culminates in thrombin formation. The positive charge on chitosan inhibited contact system activation which, in turn, suppressed the downstream coagulation cascades. Inhibition of contact activation prolongs the aPTT. In addition to positively charged amino groups, chitosan also naturally possesses hydroxyl groups along its molecular chains [33]. These may bind coagulation factors and prolong aPTT [34]. Previous studies reported that blood aPTT is prolonged in the presence of chitosan [31,35]. Kainthan et al. reported that polyglycerol-based multivalent cationic polymers do not affect PT but do prolong aPTT [36]. However, PT and aPTT only account for a small quantity of the thrombin formed during acute coagulation. The PT and aPTT assays initially evaluated how well all the coagulation factors in the coagulation cascade function together; however, the coagulation factors influenced by chitosan merit further investigation and analysis.

**Figure 9:**
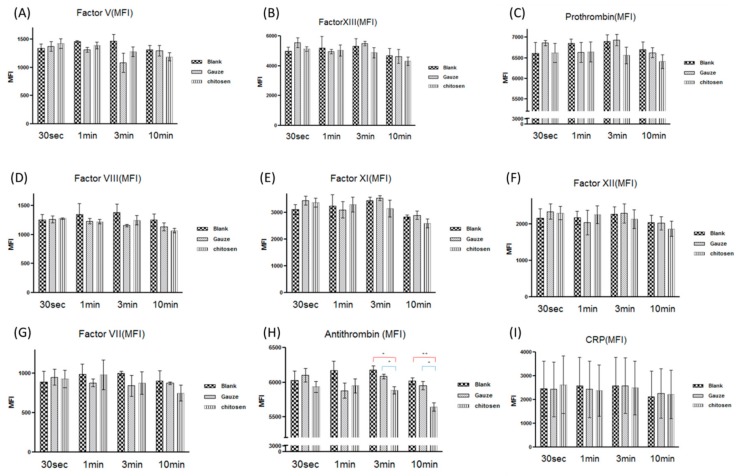
Changes in blood coagulation factor activity in response to gauze and chitosan fiber dressings. (**A**) V, (**B**) XIII, (**C**) prothrombin, (**D**) VIII, (**E**) XI, (**F**) XII, (**G**) VII, (**H**) antithrombin, and (**I**) C-reactive protein (CRP),*p<0.05, **p < 0.01.

Direct interaction between the biomaterial surface and blood affects coagulation by altering protein function in the contact activation pathway [37]. This mechanism is complex and is mediated by clotting factors I, II, V, VII, VIII, IX, X, XI, XII, and others. These represent the activities of the intrinsic, extrinsic, and common pathways in the coagulation cascade. Here, we evaluated the effects of CF dressing and gauze on coagulation proteins such as factors V, VII, VIII, XI, XII, and XIII, antithrombin, prothrombin, and C-reactive protein (CRP) (show in Figure 9A–I, respectively). Both types of dressing had similar coagulation factor activity except for antithrombin. The CF dressing significantly reduced antithrombin levels compared to the gauze dressing (Figure 9H). Chitosan is a polysaccharide with a protonated chain structure whereas the antithrombin surface is negative at pH > 5.1 [38]. We propose that chitosan may absorb and reduce antithrombin in the bloodstream via cooperative electrostatic interactions. Antithrombin or AT III inhibits several proteinases in the coagulation cascade, especially thrombin and factor Xa [39]. Factor Xa activation induces thrombin production and fibrinogen-to-fibrin conversion. The influence of chitosan on the antithrombin level may induce factor Xa to generate thrombin and accelerate coagulation. Our study demonstrated that the pro-coagulant activity of chitosan depends on a decrease in antithrombin production in the coagulation cascade.

## 4. Conclusions

The present study showed that chitosan fiber (CF) dressing had superior hemostatic properties to regular gauze-type surgical dressing. CF dressing reduced the time to hemostasis and effectively mitigated blood loss and absorption in an animal model of femoral artery hemorrhage and in patients with surgical wounds. The pro-coagulant activity of chitosan in the coagulation cascade depends on its efficacy at reducing antithrombin production. The results of this study elucidated the mechanism by which chitosan functions in hemostatic dressings. This biopolymer could be suitable as a first-line intervention for uncontrolled hemorrhage and deserves further investigation as an alternative to traditional dressings in emergency and trauma care.
